# Between medicine, humanities and the law: compiling a living archive of assisted dying

**DOI:** 10.1136/medhum-2024-012947

**Published:** 2024-06-27

**Authors:** Anna Magdalena Elsner, Vanessa Rampton

**Affiliations:** 1 University of St Gallen, St Gallen, St Gallen, Switzerland; 2 McGill University, Montreal, Quebec, Canada

**Keywords:** Law, Medical humanities, palliative care

## Abstract

Stories about personal experiences of assisted dying, a term comprising both instances when a lethal substance is administered by a physician or by the patient themselves, are frequently cited in law-making processes. These experiences of healthcare systems and the laws governing end-of-life procedures thereby interactively influence the future of medicine at the deathbed. With more countries legalising some form of assisted dying or opening political debate about the issue, addressing how these personal stories shape public opinions and social institutions is timely. In this current controversy, we question how medical humanities researchers are to make sense of the role of these stories in law-making, and critically reflect on a digital archive that seeks to make these interconnections visible. At the methodological level, the reciprocal interactions in assisted dying between medicine, law and the arts urges us to reconsider the conceptual foundations of interdisciplinary research in the medical humanities.

As much and more than a thing of the past, before such a thing, the archive should call into question the coming of the future (…) it is a question of the future, the question of the future itself, the question of a response, of a promise and of a responsibility for tomorrow.

Jacques Derrida 1998, 34–36

In 2017, the French newspaper *Libération* ran an article with the title ‘Pour une loi Anne Bert’ (In favour of an Anne Bert law), reporting on the polemical parliamentary discussions surrounding a bill meant to legalise assisted dying in France at the time. The article gathered my (AE) attention, as I recognised Anne Bert as a contemporary French author, who had described, several months earlier, in a book entitled *Le tout dernier été* (*The very last summer*), how the prohibition of assisted dying in France had led her to travel to Belgium, where she sought euthanasia following the diagnosis of a terminal illness. She had also made a documentary justifying her end-of-life choice and penned an open letter, urging all ‘citoyens libres d’une France démocratique’ (free citizens of a democratic France), to fight for a French law that would allow everyone to choose between an assisted death and palliative care. Without that choice, medicine would never be able to achieve what Bert calls ‘equality before death’ (Bert 2017, 15). As she puts it in her book, even in the French palliative care end-of-life scenario that allows for terminal sedation, it is in the end always the medical staff ‘interpreting (…) what a patient has or hasn’t said, what they want or do not want’ (Bert 2017, 15). In Bert’s plea for the right to an assisted death, her understanding of medicine plays a key role. Her book concludes by describing how, by allowing euthanasia, the Belgian healthcare system enabled her to be ‘reconciled with a kind of medicine that won’t force me anymore’ (Bert 2017, 126).

That Bert herself was engaged in a fight to change French law had been clear to me at the time, as was the fact that her own experience and understanding of medicine and how it was practised played a significant role in shaping her argument. What was news, however, and what the title of the *Libération* piece reflected, was that Bert’s call for legal change and rethinking the role that medicine should play in dying had found a prominent place in law-making processes. A search of the *Assemblée nationale* (French Parliament) records reveals that between 2017 and 2023, Bert’s legacy was cited in several parliamentary debates and meetings alongside famous French end-of-life ‘cases’, such as Vincent Lambert or Chantal Sebire, to highlight why a change in the law was needed. Indeed, Bill 288 (*Proposition de loi donnant le droit à une fin de vie libre et choisie*) (Bill on the Provision of a Free and Chosen End of Life), which the *Libération* piece referred to, not only cites Bert verbatim, but also explains that she is one of the coauthors of the bill. In other words, not only Bert’s particular ‘case’—the fact that she was diagnosed with a terminal condition and travelled to another jurisdiction to seek a medically assisted death—but how she herself framed that case in a kaleidoscopic multimedia approach, had in no small way triggered and shaped a law-making process. As part of this, the disparate elements of her *Gesamtkunstwerk* centring on her choice to die an assisted death became themselves inscribed in that process.

While thinking about how to make sense of the status and role of Bert’s cultural productions for assisted dying law-making in France, I also wondered what kind of research approach could address this complex entanglement of medicine, law and the arts. Over time, it became increasingly clear to me that Bert is just one among many writers, filmmakers and artists, whose activist work based on personal stories navigated a similar medicolegal trajectory. The French film *Intouchables,* by Nakache and Toledano (2011), one of the most successful French films in and outside of France, has a transnational legacy in that regard, being cited several times in the Bundestag debates on the question. *Mar adentro*, by Alejandro (2004), a Spanish film on the assisted death of Ramón Sampedro, featured consistently in the debates prior to the introduction of the 2021 euthanasia law in the country, something that was acknowledged by medical journalism (Casino 2004). In Germany, the fictional play *Gott*, by Von Schirach (2020), was adapted specifically to debates in the Bundestag about physician-assisted dying, and was, in turn, recommended by the German law-making body as a fictional work of relevance to the issue at hand. In time, my growing interest in these potentially relevant narrative works became such a long list, a kind of alternative archive that ran parallel to the archives of parliamentary debates, that it took on a life of its own as a living, changing collection of sources that was being constantly fed by recommendations from others.

The desire to come to grips with the epistemic status and moral weight of such stories for law-making processes was the basis of a set of academic questions and the application for a multiyear, international research project. European Research Council (ERC) funding aims to support early career researchers, while also encouraging them to step out of their comfort zones. When I submitted the project, a key aim was to show how the study of films and texts—the familiar material of the humanities—could inform those interested in understanding how legal change occurs, and the way in which it shapes what medicine can and cannot do at the end of life. This required comprehending how the insights of the medical humanities—the various cultural taboos surrounding death, the effects of the representation of death on screen, for example—complement or intersect with existing approaches in legal studies. As a medical humanities scholar, the study of law was a new endeavour for me, and it required that I come to grips with the existing ways in which law engages with the humanities, and popular culture in particular (Sherwin 2000). Within legal scholarship, the reliance of law-making processes on and their entanglement with texts and films is not rejected outright, but rather relegated to that corner of legal studies concerned with ‘law and culture’ or ‘law and humanities’. The desire to place activist writing and filmmaking front and centre within legal processes was, therefore, a high-risk/high-reward endeavour, also due to potential resistance from legal scholarship.

The disciplinary configuration required to study the role of assisted dying narratives in law and medicine thereby also challenged what we may think of as the interdisciplinary or transdisciplinary heart of the medical humanities. This is because of the kind of circular configuration that Bert’s example illustrates: a (literary) text is informed by a medical experience and positions medicine in a certain way, and the (literary) text about assisted dying is cited in a legal context. This recounting of a medical experience in the text may, in turn, be instrumentalised to shape the legal role that medicine can play in the future of dying. What was needed is best captured by what Marco Biagioli has called ‘post-disciplinary liaisons’, namely a focus on research questions rather than disciplines. Such a focus may lead to temporary configurations or ‘problem-specific collaborations’ (Biagioli 2009, 821), a kind of ‘assemblage of very diverse knowledge-making practices’ (Biagioli 2009, 824). I came up with what I viewed as such an ‘assemblage’ and what I called at the time of writing the grant proposal, ‘an online, open access annotated bibliography and filmography on assisted dying’, which aims to generate research and teaching with regard to assisted dying, and which would be continuously updated throughout the project. Constantly updating this bibliography, I wrote, will be key for establishing a dialogue with law-making procedures and tracing the culturally specific ways in which end-of-life medicine is approached and practised. The platform would provide, first, a record of the primary sources and stories that influenced law-making processes, as well as, second, an opportunity to reflect on those processes by keeping conscious track of how an archive is produced, and the limitations, biases and uncertainties built into that process.

In 2022, the project was formally awarded 5-year ERC funding, which facilitated the establishment in 2023 of a small research team with backgrounds in literature, law and philosophy investigating how the stories of assisted dying both influence the legislative contexts in which they are written and are shaped by the contexts themselves. As an intellectual historian, I (VR) joined the project and took on the role of working on the conceptual connections between medicine, stories and the law, as well as coordinating the online open-access repository. This repository soon turned into the project of creating a website together with designers and programmers, who were willing and able to engage with the theoretical and ethical exigencies of portraying the stories of assisted dying on the web. The website, available at assistedlab.org, increasingly grew into a core part of the project and now employs three research assistants with backgrounds in legal and literary studies who work exclusively on what we now refer to as ‘the archive’. Our terminology shifted from ‘online, open-access bibliography’ to ‘the platform’, ‘the database’, ‘the repository’, and ended up at ‘the archive’ because what had started out in the project proposal as a preliminary corpus of approximately 60 texts and 45 films from France, Germany, Belgium, Switzerland and the Netherlands since 2000, soon took on a different dimension. Only a couple of months into the project, we already had over 300 references to texts, films, blogs, performances, short videos and audio recordings that had figured in assisted dying law-making processes in over 15 countries. With many countries debating or approving legalisation, the tendency was—and continues to be—growing.

As our research agenda consolidated, the next logical step was turning what we had discovered into a searchable archive, paying particular attention to how we collect sources, document them and make them accessible. At the same time, it was clear that this archive would not only enable us and others to study the history of assisted dying through cultural productions, but would also be an archive of the present and the future. Any cultural production about assisted dying—such as the Academy-award winning Canadian film *Les invasions barbares*, by Denys Arcand (2003)—was first a ‘film about (assisted) dying’ before—in the case of this particular film—being referenced multiple times in the Québec parliament a decade later. The film thereby became part of the argumentative strategy that led to assisted dying’s legalisation in Québec (2015) and in Canada (2016), even if the fact that it would be heavily cited in parliament was not foreseeable when it first came out. Our experience of discovering references to cultural productions in law-making processes while finding many more such productions not yet referenced in law-making documents, but that could be in the future, is probably best described by what the French philosopher Jacques Derrida has called ‘archive fever’. He captured its acute state in the following terms: ‘It is to burn with a passion. It is never to rest, interminably, from searching for the archive, right where it slips away. It is to run after the archive, even if there’s too much of it, right where something in it anarchives itself’ (Derrida 1998, 91). Unlike the Derridean scenario, where the search for the *arche*, the beginning and therefore the idea of understanding the present via the past is key, our emerging archive was based on a different approach to knowledge. As an archive of the very recent past but mainly the present, its findings delineated potential future legal landscapes in which medicine at the end of life was assigned a specific place. In other words, our archive was itself generating and developing potential stories of the present and the future of medicine.

The growing list of possible entries—and the questions they raised—at times overwhelmed us, but also allowed us to realise that methodologically speaking, this digital, cultural and continuously expanding archive was the first step to everything. It provided sources that illustrate how rights claims were intertwined with personal experiences of medicine, it documented and reinforced our collective sense that cultural productions were actively being referenced in legal settings, it assured the project’s visibility and possible interactions with lawmakers, as well as its potential use for teaching and research, and it forced us to consider—through how we chose and positioned the often activist stories—our own identity as an academic, non-partisan research lab. It also very much put us out of our comfort zone. That is because the research questions we asked were no longer the familiar ones of cultural scholarship (how is assisted dying depicted in x and why?). They also exceeded the ones we associate with the medical humanities (what role does medicine play in a text or film about assisted dying?). The archive is an emerging intersection between the arts, law and medicine that pushed us into unfamiliar territory.

Indeed, from the time we as a research team began thinking about the archive, rather than a set, well-defined product, we had uncertainty, openness and biased human choices. Rather than stability, cumulation and description, we had fluidity, blank spots and normativity. In short, rather than an objective, easy-to-delimit archive/product, we had questions that forced us to scrutinise the rationales of our archival principles and practices. For example, as researchers, our aim in creating this archive is not to advocate for or against (medically) assisted dying. Instead, we seek to describe contemporary cultural practices and to reveal insufficiently noticed links between stories and legal change. However, every description has its own kind of normativity. In highlighting the extent to which stories matter—as well as the fact that some matter more than others—we aim to shed light on the importance of narratives for coming to grips with legal transformations that change the role and the goals of medicine. By highlighting the plurality of stories of assisted dying, and the fact that they hold no single message for those in favour of the legalisation of the practice, we also aim to show the complexity of the stories describing an event as existentially significant as an assisted death.

Both law and medicine shape the public sphere and yet they are driven by and feed back to the individual, private figure. A key component of how individuals respond to and influence legal regulations, and the place medicine is assigned within them, is communicated via aesthetic form. Unlike oral histories or empirical research relying, for example, on interviews with those choosing an assisted death, the stories collected in our archive have all been intentionally, aesthetically arranged and are therefore governed by their creators’ own logics and rhetoric. These complex aesthetic choices are shaped by self-fashioning as much as cultural practices around the good death and the culture industry at large. By being referenced in law-making documents, these aesthetics are shaping the future of legal imaginations around assisted dying. That is, law-making procedures have woven these stories into the fabric of the laws they set out to make without overtly recognising and describing this material as a specific form of evidence—neither testimonial, nor case, but rather stories that function in a particular way. Our digital, cultural archive provides a forum for us to address both the conscious and unconscious processes that influenced its compilation, and in doing so to reflect on the ethics of knowledge creation and dissemination more broadly. In essence, the genesis of our archive is the high-risk endeavour of merging digital with medical and legal humanities. As such, the archive also lays open some of the challenges arising in ‘postdisciplinary liaisons’ and as such constitutes a logbook for other researchers engaging with projects that require us to face the adventures associated with moving beyond the silos of disciplinary and interdisciplinary knowledge creation.
